# Non-Volatile Taste Profile Dynamics Across Developmental Stages of *Agaricus bisporus* Fruiting Bodies

**DOI:** 10.3390/foods15132375

**Published:** 2026-07-03

**Authors:** Lingzhong Wan, Hongjuan Wang, Sheng Liu, Ying Ni, Xiaonan Deng, Xiaoming Yan, Changjiu Tian, Qianwen Li, Jiabao Zhu

**Affiliations:** 1 Institute of Industrial Crops, Anhui Academy of Agricultural Sciences, Hefei 230001, China; wan520lz@mail.ustc.edu.cn (L.W.); whj14725@163.com (H.W.); ny18895329564@163.com (Y.N.); xn_deng@foxmail.com (X.D.); 19909699660@163.com (X.Y.); changjiutian@163.com (C.T.); 2 Edible and Medicinal Mushroom Innovation Centre, Anhui Academy of Agricultural Sciences, Hefei 230001, China; 3 College of Food Science and Engineering, Shandong Agriculture and Engineering University, Jinan 250100, China; z2023066@sdaeu.edu.cn; 4 School of Materials and Chemistry, Anhui Agricultural University, Hefei 230036, China

**Keywords:** non-volatile taste profile, *Agaricus bisporus*, fruiting body, non-targeted metabolomics, tissue, accumulation pattern

## Abstract

Beyond nutrition, taste quality is a key quality trait driving the global popularity of *Agaricus bisporus*. This study systematically investigated non-volatile taste-related metabolite dynamics in caps and stipes during fruiting body development using non-targeted metabolomics. Among 1358 identified metabolites (974 in caps, 997 in stipes), 328 taste-related metabolites were screened. Applying screening criteria of VIP > 1, *p* < 0.01, and fold change ≥ 2 or ≤ 0.5, 492 and 446 differentially accumulated metabolites (DAMs) were identified in cap and stipe during fruiting body development, respectively. Cross-tissue comparison revealed 975 tissue-specific DAMs between cap and stipe across all developmental stages. Notably, 127 and 116 taste-related DAMs in cap and stipe, respectively, exhibited seven distinct accumulation profiles. Key umami-related compounds, aroma precursors, and antioxidants peaked in cap tissue at stage 3 (closed cup stage), suggesting a preliminary optimal harvest timing for market-quality mushrooms based on metabolic profiling of non-volatile taste-active compounds. Organic acids and nucleotides were more abundant in immature stages, while phosphorylated six-carbon sugars showed stipe-dominant accumulation at middle–late stages. Notably, all taste-related conclusions are inferred from non-volatile metabolite characterization rather than direct sensory measurements. KEGG pathway enrichment highlighted that taste-related metabolites primarily shaped taste via amino acid biosynthesis, cofactor metabolism, lysine biosynthesis, and nucleotide pathways. These insights provide a metabolic foundation for optimizing cultivation strategies and enhancing product quality in *Agaricus bisporus*.

## 1. Introduction

*Agaricus bisporus* (*A. bisporus*) is the most extensively cultivated mushroom worldwide, contributing approximately 30% to global mushroom production and enjoying widespread popularity. First cultivated in 18th-century Paris, this “world mushroom” represents a landmark success in the artificial cultivation of edible fungi [1]. Numerous studies demonstrate that *A. bisporus* is a highly nutritious edible mushroom. It is rich in high-quality protein and all essential amino acids, with cell walls containing chitin, trehalose and β-glucan that serve as functional dietary fibers. This fungus is also abundant in B vitamins, vitamin D, potassium, phosphorus, iron, zinc and selenium. Characterized by low fat and a high proportion of unsaturated fatty acids, it has become an ideal healthy food and a good substitute for animal protein [2,3]. Beyond its high nutritional value, taste is a key quality trait driving its broad consumption. The characteristic taste of *A. bisporus* is largely determined by non-volatile taste components, including soluble sugars, polyols, organic acids, free amino acids, and 5′-nucleotides [4]. Among these, 5′-nucleotides and taste-active amino acids synergistically enhance umami, and their varied compositions and ratios underpin mushroom taste diversity [5,6]. Taste peptides further strengthen umami intensity, acting both as direct taste enhancers and precursors of volatile aroma compounds [7,8]. Existing research indicates that strain, cultivation practice, substrate, and developmental stage significantly influence the biosynthesis and accumulation of taste-related metabolites [9,10,11]. Systematic profiling of these non-volatile compounds is thus critical for clarifying the taste formation mechanism of edible mushrooms, supporting the development of mushroom-based flavorings and enhancing the commercial value of *A. bisporus*.

High-performance liquid chromatography (HPLC) is the traditional analytical method for the quantitative determination of taste components in mushrooms. This technique enables the accurate quantitative detection of major taste-active compounds, such as free amino acids and 5′-nucleotides, in *A. bisporus* at different maturity stages [12,13,14]. However, HPLC has an inherent limitation: its narrow metabolite coverage restricts the comprehensive characterization of the overall flavor profile of *A. bisporus*. In contrast, metabolomics based on ultra-high-performance liquid chromatography–tandem mass spectrometry (UPLC-MS/MS) has emerged as a powerful analytical platform for metabolomic profiling, featuring high throughput, superior sensitivity, and broad metabolite coverage. Recent metabolomic studies on edible fungi have identified key taste compounds and related metabolic pathways in morels, truffles, and boletes [5,15,16]. Additionally, combined transcriptomic and metabolomic analyses have revealed that differences in organic acid content drive the taste divergence between wild and cultivated *A. bisporus* [11]. These studies have validated the applicability of metabolomics in fungal taste research; however, they lack a systematic analysis of the dynamic changes in non-volatile taste compounds throughout the entire developmental process of *A. bisporus* fruiting bodies. Moreover, few studies have clarified the tissue-specific accumulation characteristics of taste metabolites (i.e., cap vs. stipe) and the underlying molecular mechanisms regulating such specificity. Clarifying the dynamic changes and tissue specificity of taste-related metabolites during *A. bisporus* development is crucial for determining the optimal harvest stage, improving cultivation strategies, and breeding high-taste mushroom strains.

**Figure 1:**
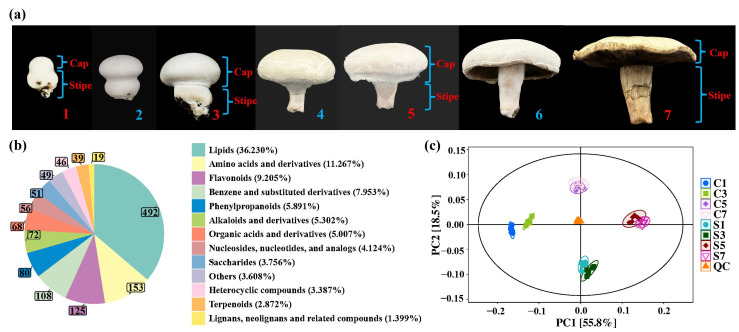
(**a**) Seven different development stages of *A. bisporus* fruiting bodies (1: “Pinhead” stage; 2: “Button” stage; 3: “Closed cup” stage; 4: “Cup1” stage; 5: “Cup 2” stage; 6: “Flat1” stage; 7: “Flat 2” stage). The stages with red font were selected for metabolomic analysis and were respectively named as stage 1, stage 3, stage 5 and stage 7. (**b**) The proportion of identified metabolites within each chemical classification. Distinct color blocks correspond to different chemical classifications. (**c**) The principal component analysis score plot of cap and stipe tissues of *A. bisporus* fruiting bodies during different developmental stages. (C1: cap, stage 1; C3: cap, stage 3; C5: cap, stage 5; C7: cap, stage 7; S1: stipe, stage 1; S3: stipe, stage 3; S5: stipe, stage 5; S7: stipe, stage 7; QC: quality control sample; PC1: principal component 1; PC2: principal component 2.).

In this study, we aimed to investigate the dynamic accumulation patterns of non-volatile taste compounds and explore the potential taste development mechanism during the fruiting body development of *A. bisporus*. Four representative developmental stages (stage 1, stage 3, stage 5, and stage 7) were selected from the seven classic developmental stages of *A. bisporus* fruiting bodies (Figure 1a), and samples from different tissues (cap and stipe) at these stages were used as experimental materials. Non-targeted metabolomics was employed to analyze the metabolites in the cap and stipe at the four developmental stages. Furthermore, the dynamic variations in amino acids and their derivatives, nucleosides and nucleotides, organic acids, and saccharides—key substances regulating taste formation during mushroom development—were elucidated. Combined with metabolomic pathway analysis, the dominant metabolic pathways of taste-related substances in *A. bisporus* were tentatively explored. In addition, differential metabolomic profiles between cap and stipe tissues at the four growth stages were further analyzed. This study provides new insights into the tissue-specific taste profiles and the mechanisms underlying mushroom taste development, laying a theoretical foundation for the targeted enhancement of *A. bisporus* taste.

## 2. Materials and Methods

### 2.1. Chemical Reagents

All chromatographic-grade chemical reagents were used in this study. Acetonitrile (ACN), methanol (MeOH) and 2-propanol (IPA) were purchased from CNW Technologies (Dusseldorf, Germany); acetic acid was from Sigma-Aldrich Chemical Co. Ltd. (St. Louis, MO, USA). Deuterated standards included decanoic-d19 acid (≥98%) and trans-zeatin-d5 (≥98%) (Shanghai Bvant Biotechnology Co., Ltd., Shanghai, China), as well as diisobutyl phthalate-3,4,5,6-d4 (99.9%), indole-3-acetic-2,2-d2 acid (99.0%) and glycocholic acid-d4 (99.7%) (Beijing Mahage Biotechnology Co., Ltd., Beijing, China). Deionized water was obtained from Watsons (Hong Kong, China).

### 2.2. Mushroom Materials

First-flush *A. bisporus* at different developmental stages were collected from Funan Lian Mei Agricultural Products Co., Ltd., Fuyang, China, a local commercial farm with standardized cultivation (uniform strain, substrate, temperature, humidity and ventilation). The factory cultivation of *A. bisporus* strictly complied with Anhui Provincial Local Standard DB34/T 4220-2022 Technical regulations for the factory production of *Agaricus bisporus* [17]. The main culture substrate formula was composed of 53% wheat straw, 43% chicken manure, 1% gypsum powder, 2% lime and 1% superphosphate. The water content of the prepared compost was controlled at 70–75% with an initial pH value of 8.0–8.5. The compost underwent two-stage tunnel fermentation and spawn running fermentation successively following standard temperature and humidity protocols. The sowing rate was controlled at 0.5 kg/m^2^–1.0 kg/m^2^. High-quality peat soil was used for casing, with casing thickness maintained at 4–5 cm, water content of 68–72% and pH value adjusted to 7.5–8.0. During the mycelium running stage, the mushroom house temperature was set at 22–26 °C and relative air humidity at 75–80%. For fruiting management, the temperature was controlled at 16–18 °C, relative air humidity at 80–85%, and carbon dioxide concentration maintained between 1000 mg/L and 1500 mg/L. All cultivation parameters remained consistent throughout the whole production cycle. Sampling from a single farm eliminated metabolic profile interference from heterogeneous cultivation conditions, ensuring reliable analysis of taste-related metabolite dynamics during fruiting body development. *A. bisporus* development was artificially divided into seven stages per Hammond et al. [18] (Figure 1a). In commercial factory cultivation, the entire developmental process from stage 1 to stage 7 finished within approximately 18 days after casing, and stage 1 (pinhead primordia) firstly appeared on the about 14th day post-casing. Considering the fluctuation of growth rate caused by environmental factors, we defined each developmental stage mainly based on fruiting body morphology and cap diameter for accurate staging and harvesting guidance [18]. Four stages (1, 3, 5, 7, red-labeled in Figure 1a) were selected for metabolomics, representing distinct morphological characteristics and developmental transitions from immature pinhead to fully mature flat fruiting bodies. This selection captured taste-related metabolite dynamics across key developmental processes while avoiding redundant sampling. Six biological replicates were collected for each stage; fruiting bodies were dissected into cap and stipe, and tissues were immediately frozen in liquid nitrogen.

### 2.3. Metabolite Extraction

Mushroom tissues were lyophilized using a vacuum freeze-dryer (Beijing Sihang Qihang Co., Ltd., Beijing, China) prior to grinding. The lyophilization conditions were as follows: pre-freezing at −80 °C overnight, condenser temperature of −87.3 °C, sample temperature during drying of −11.6 °C, vacuum pressure of 0.9 Pa, and total drying time of 48 h. Afterwards, the dried samples were ground to a fine powder, and 25 ± 1 mg aliquots were transferred to EP tubes under low temperature. Homogenization beads and 1000 μL extraction solution (MeOH:ACN:H_2_O, 2:2:1, *v*/*v*/*v*) containing deuterated internal standards (glycocholic acid-d4, 0.09 μmol/L; diisobutyl phthalate-3,4,5,6-d4, 3.5 μmol/L; decanoic-d19 acid, 0.16 μmol/L; trans-zeatin-d5, 0.5 μmol/L; indole-3-acetic-2,2-d2 acid, 2.4 μmol/L) were added, and the mixture was vortexed for 30 s. Samples were homogenized (35 Hz, 4 min) and sonicated in an ice bath at 4 °C for 5 min, with this cycle repeated three times. Protein precipitation was performed by incubation at −40 °C for 1 h, then samples were centrifuged at 12,000 rpm (13,800× *g*, 8.6 cm radius) at 4 °C for 15 min. The supernatant was filtered through a 0.22 μm membrane and transferred to glass vials for UPLC-MS/MS analysis. An equal volume of supernatant from each sample was pooled to prepare quality control (QC) samples.

### 2.4. UPLC-MS/MS Analysis

UPLC-MS/MS analyses were performed on a Vanquish UHPLC system (Thermo Fisher Scientific, Waltham, MA, USA) coupled with an Orbitrap Exploris 120 mass spectrometer (Thermo, Waltham, MA, USA), using a Phenomenex Kinetex C18 column (Phenomenex, CA, USA; 2.1 mm × 50 mm, 2.6 μm). Mobile phase A was 0.01% acetic acid in water, and mobile phase B was isopropanol/acetonitrile (1:1, *v*/*v*), with a constant flow rate of 0.30 mL/min. The elution gradient for phase B was: 1% (0.00–0.50 min), linear increase to 99% (4.00 min) and hold (4.00–4.50 min), reversion to 1% at 4.55 min and hold until 6.00 min. The autosampler was maintained at 4 °C with an injection volume of 2 μL. Full-scan and MS/MS spectra were acquired in information-dependent acquisition (IDA) mode via Xcalibur 4.4 (Thermo Scientific, Waltham, MA, USA). ESI ion source parameters were: sheath gas 50 Arb, auxiliary gas 15 Arb, nebulizer temperature 350 °C, capillary temperature 320 °C; full-scan MS resolution 60,000, MS/MS resolution 15,000, stepped normalized collision energy (SNCE) 20/30/40. Spray voltage was 3.8 kV (positive ion mode) and −3.4 kV (negative ion mode). Analytical conditions were based on established UPLC-MS/MS metabolomic methods for edible fungi [19].

### 2.5. Data Processing and Statistical Analysis

The raw data were converted to mzXML format via ProteoWizard (V3.0.24054) and processed with an in-house R program built on XCMS (V4.1.12) for peak detection, extraction, alignment and integration. Metabolite identification was performed using R packages (ggplot2, V3.3.5) and BiotreeDB (V3.0), with annotation confidence graded per the Metabolomics Standards Initiative (MSI) [20] and the definitions of Zhou et al. [21]: Level 1 (in-house standard matching, MS1 + RT + MS/MS); chiral metabolites (amino acids, saccharides, etc.) had stereochemical configurations confirmed via chiral standard matching to ensure accurate annotation of biologically active isomers (L-amino acids, D-saccharides). Level 2 (standard MS/MS library matching, MS1 + MS/MS, no RT); chiral metabolites in this level had configurations inferred based on dominant fungal isomers and reported MS/MS characteristics, restricted to those with documented biological relevance in *A. bisporus* or related edible fungi. Only Level 2 and higher metabolites were included in subsequent analyses, with MS2 scores calculated per Zhou et al. [21].

Prior to statistical analysis, missing values were imputed as half the minimum value for each metabolite, followed by internal standard normalization to reduce technical variability. The normalized dataset (peak numbers, sample names, normalized peak areas) was imported into SIMCA 18.0.1 for multivariate analysis, including PCA and OPLS-DA for sample distribution and grouping visualization. Differential accumulated metabolites (DAMs) were screened by VIP > 1, *p* < 0.01 (Student’s *t*-test) and FC ≥ 2 or ≤0.5. Heat maps, Venn plots and K-means clustering were constructed using R packages, including R (cluster, V2.1.0) for K-means clustering, R (VennDiagram, V1.6.20) for Venn plots, and R (pheatmap, V1.0.12) for heatmaps. All metabolites were annotated against the KEGG Compound Database with “*Agaricus bisporus var. bisporus H97”* as the reference; KEGG pathway enrichment analysis of DAMs was conducted via hypergeometric test, with statistical significance defined as *p* < 0.05.

## 3. Results and Discussion

### 3.1. Metabolite Analysis in Cap and Stipe Across Growth Stages

*A. bisporus* fruiting bodies were harvested at stages 1, 3, 5 and 7, with cap and stipe tissues subjected to UPLC-MS/MS analysis. A total of 1358 metabolites were identified in all samples (Table S1), including 974 in caps and 997 in stipes; 361 and 384 metabolites were unique to caps and stipes, respectively, with 613 shared between the two tissues (Figure S1). Statistical classification assigned all metabolites to 13 categories (Figure 1b): lipids (492), amino acids and derivatives (153), flavonoids (125), benzene and substituted derivatives (108), phenylpropanoids (80), alkaloids and derivatives (72), organic acids and derivatives (68), nucleosides, nucleotides, and analogs (56), saccharides (51), heterocyclic compounds (46), terpenoids (39), lignans, neolignans and related compounds (19), and others (49, primarily aldehydes, ketones, amides, alcohols). Detailed metabolite numbers and proportions per class in cap and stipe tissues are shown in Figure S2.

Multivariate statistical analysis was performed to characterize metabolic variations in cap and stipe across growth stages. Principal component analysis (PCA; Figure 1c) showed the first two components explained 74.3% of total variance; tight clustering of quality control (QC) samples confirmed analytical stability and experimental reproducibility, with QC deviations within 2 SD and temporal variations acceptable for semiquantitative analysis (Figure S3). Eight sample groups were clearly separated, with cap and stipe groups forming distinct clusters along the PCA diagonal (reflecting tissue-specific metabolic divergence); partial overlaps between C5–C7, S1–S3 and S5–S7 indicated transitional metabolic states. Orthogonal partial least squares discriminant analysis (OPLS-DA) models were constructed for sequential stage comparisons (C3 vs. C1, C5 vs. C3, C7 vs. C5; S3 vs. S1, S5 vs. S3, S7 vs. S5) to enhance low-correlation variable sensitivity. Score plots (Figure S4) robustly separated paired groups, validating stage-specific metabolic phenotypes; model reliability was confirmed by 200 permutation tests (Figure S5), with all Q^2^ intercepts < 0 ruling out overfitting. These results aligned with PCA and confirmed OPLS-DA efficacy for resolving subtle metabolic changes during fruiting body development.

In accordance with MSI identification criteria, 328 taste-related metabolites (amino acids and derivatives, nucleosides, nucleotides, and analogs, organic acids and derivatives, saccharides) were screened from Level 1/2 metabolites. Level 1 metabolites (core for taste analysis, absolute qualitative/quantitative accuracy) were identified via MS1 exact mass, retention time and MS/MS fragmentation matching with in-house standards (L-glutamate, L-ergothioneine, citric acid, inosine, etc.). Level 2 metabolites were annotated by MS1 exact mass and MS/MS fragmentation matching with standard libraries (3-aminobutanoic acid, α, α’-trehalose 6-phosphate, UDP-xylose, etc.). All Level 1/2 taste-related metabolites were included in subsequent differential accumulation and K-means clustering analyses to ensure comprehensive, reliable taste profile investigation.

### 3.2. DAM Screening in Cap and Stipe During Fruiting Body Development

**Figure 2:**
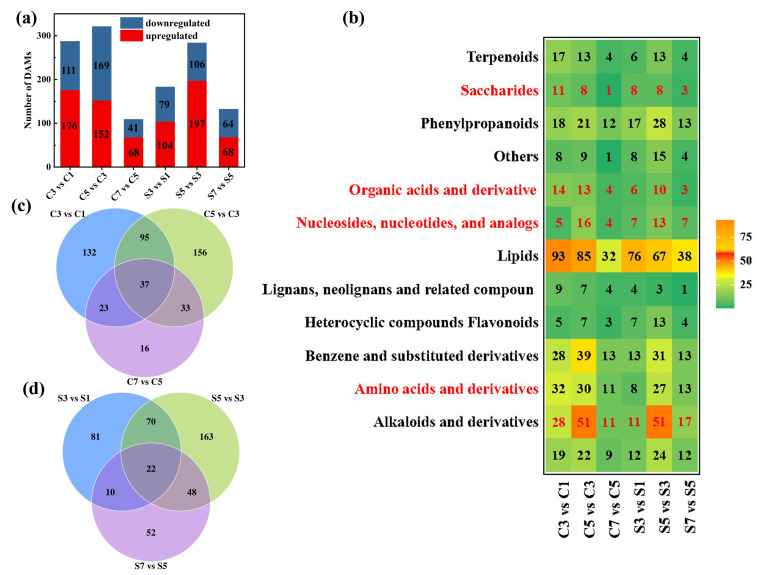
(**a**) Numbers of upregulated and downregulated DAMs in each paired comparison (C3 vs. C1, C5 vs. C3, C7 vs. C5, S3 vs. S1, S5 vs. S3, and S7 vs. S5); (**b**) chemical classification of DAMs in pairwise comparisons; (**c**) Venn diagram showing the number of DAMs in the cap comparison groups; (**d**) Venn diagram showing the number of DAMs in the stipe comparison groups.

DAMs in cap and stipe between adjacent growth pairs were screened based on the OPLS-DA model using VIP >1, *p* < 0.01 and FC ≥ 2, or ≤ 0.5. A total of 492 and 446 DAMs were respectively screened from each cap comparison (C3 vs. C1, C5 vs. C3, C7 vs. C5) and each stipe comparison (S3 vs. S1, S5 vs. S3, S7 vs. S5) (Tables S2–S7). These metabolites mainly fall into categories such as lipids, amino acids and derivatives, organic acids and derivatives, flavonoids, benzene and substituted derivatives, nucleosides, nucleotides, and analogs in both cap and stipe. Figure 2a depicts the number of tissue-specific DAMs between adjacent growth stages of fruiting bodies. The comparison revealed that the early growth stages (pinhead and closed up stages) regulated 287 and 183 metabolites in cap and stipe (C3 vs. C1, S3 vs. S1), respectively. Then, the greatest numbers of DAMs in cap and stipe tissues were both observed in group stage 5 vs. 3 (C5 vs. C3, S5 vs. S3). Upregulated metabolites exhibited significantly higher levels in the target stage compared to the reference stage, with a FC > 2, VIP > 1, and *p* < 0.01. Conversely, downregulated metabolites displayed a significant reduction in the target stage relative to the reference stage, meeting the thresholds of FC < 0.5, VIP > 1, and *p* < 0.01. In the cap, 152 DAMs were upregulated and 169 were downregulated. In the stipe, 197 DAMs were upregulated and 106 were downregulated. Metabolite changes gradually decreased during subsequent Flat 2 vs. Cup 2 stage (stage 7 vs. 5), with 109 and 132 metabolites regulated in cap and stipe, respectively. The clustered heatmaps of the top 20 DAMs that are significantly upregulated and downregulated for pairwise comparisons are shown in Figure S6.

Figure 2b shows the chemical classification of DAMs in pairwise comparisons from adjacent growth stages. We focused on the changes in taste-related metabolite during the fruiting body development, which including amino acids and derivatives, nucleosides, nucleotides, and analogs, organic acids and derivatives, and saccharides. Notably, amino acids and derivatives (51 in cap, 51 in stipe), nucleosides, nucleotides, and analogs (16 in cap, 13 in stipe), organic acids and derivatives (13 in cap, 10 in stipe), saccharides (8 in cap, 8 in stipe) were predominant in stage 5 vs. 3, suggesting the critical growth stage in taste formation. The Venn diagram depicts tissue-specific common and unique metabolites across different growth stages in the cap and stipe, respectively (Figure 2c,d). A total of 37 and 22 common key differentially accumulated metabolites (DAMs) in the cap and stipe underwent continuous changes throughout the development process (Figure S7). These findings underscored tissue-specific metabolic reprogramming during development, with amino acids, nucleosides, nucleotides, and organic acids as key determinants of *A. bisporus* quality and taste [22,23].

### 3.3. Changes in DAMs in Cap and Stipe During Fruiting Body Development

**Figure 3:**
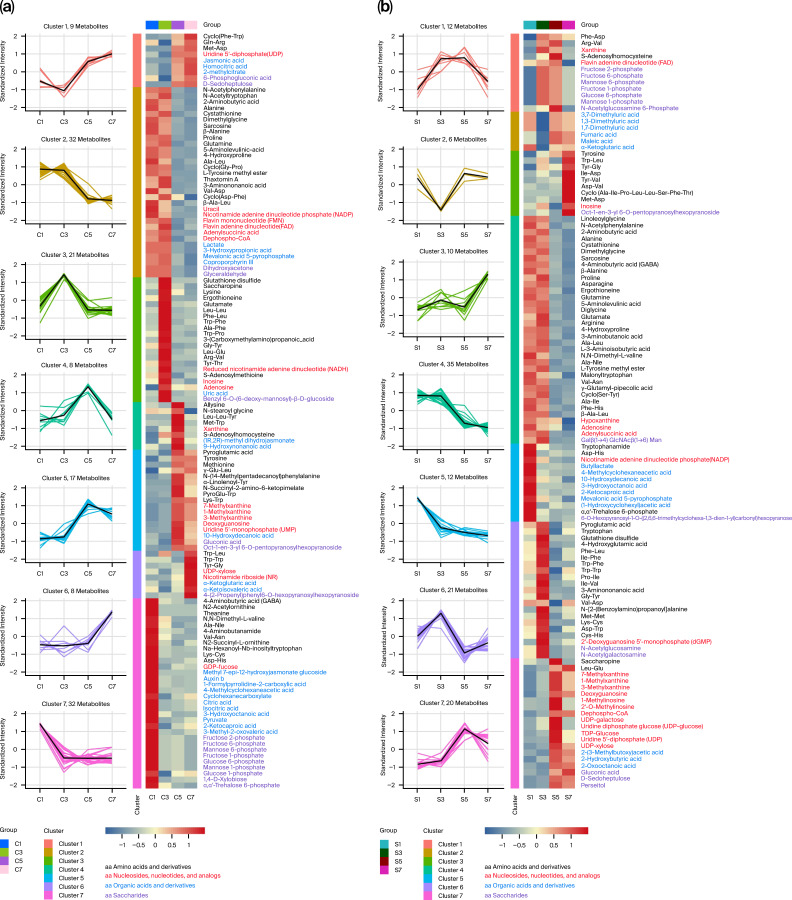
The K-means clustering analysis on taste-related DAMs in (**a**) cap and (**b**) stipe during fruiting body development. The line charts show the z-score standardized content of taste-related DAMs in each cluster; the corresponding heatmap shows the normalized abundance of taste-related DAMs.

The fruiting body development is associated with metabolite accumulation, which leads to changes in the appearance, texture, and taste of the mushroom [15]. To visualize the trend of taste-related DAM changes in cap and stipe during fruiting body development, K-means clustering and heatmap analysis were employed to analyze the relative content of DAMs after standardization (Figure 3).

#### 3.3.1. Amino Acids and Derivatives

Amino acids and derivatives are key components of both nutrition and taste mushrooms. They encompass free amino acids (e.g., aspartate and glutamate, vital for umami perception), modified forms, and oligopeptides [24]. Their taste profiles in *A. bisporus* are determined by structural features (hydrophobicity, carbon chain size, charge, side-chain functional groups, α-carbon chirality) that mediate taste receptor interactions [25,26]. In the cap, 67 differential amino acids and derivatives clustered into seven groups (Figure 3a). Cluster 1 (3 oligopeptides) showed a biphasic trend (decline stage 1–3, then increase), indicating stage-specific enzymatic processing. Cluster 2 (20 compounds, e.g., N-acetyl amino acids, sweet alanine/proline, umami glutamine) declined stepwise; N-acetyl amino acids enhance saltiness, umami, kokumi and lingering taste [27]. Cluster 3 (16 compounds) peaked at stage 3 (redox-related glutathione disulfide, GSSG; umami glutamate; antioxidant ergothioneine, EGT; methyl donor S-adenosylmethionine, SAM; bitter lysine), marking stage 3 as metabolically optimal for commercial harvesting. Notably, the peak abundance of SAM at stage 3 is metabolically coupled to the biosynthesis of sulfur-containing volatile organic compounds (VOCs), which dominate the characteristic aroma of edible mushrooms [28]. As the principal activated methyl donor in fungal sulfur metabolism, SAM provides methyl groups for transmethylation reactions in the methionine–cysteine pathway, the key route for generating dimethyl sulfide (DMS), dimethyl disulfide (DMDS), dimethyl trisulfide (DMTS) and other sulfur volatiles. High SAM levels at stage 3 boost the activity of cysteine-S-conjugate beta-lyase and S-adenosylmethionine-dependent methyltransferases, driving the transformation of non-volatile sulfur amino acid precursors into aroma-active volatiles [29]. This precursor–product relationship confirms that stage 3 coincides with the strongest potential for both umami taste formation and characteristic volatile aroma synthesis, further validating stage 3 as the preferred harvest stage. The glutathione, glutathione (GSH)/GSSG ratio modulates umami synthesis and GSH improves mouthfeel [30]. Clusters 4–5 (5/9 compounds, peak stage 5) featured S-adenosylhomocysteine, SAH (SAM methylation byproduct), which redirects flux to sulfur flavor precursors and supports GSH/EGT biosynthesis, with SAM/SAH showing an inverse relationship (SAM peak stage 3, SAH peak stage 5) [31]; this cluster also included bitter tyrosine, sweet methionine and umami/astringent pyroglutamic acid/γ-Glu-Leu [32,33,34]. Cluster 6 (three oligopeptides) was stable at stage 1–5 then sharply increased. This pattern might suggest a delayed but accelerated enzymatic hydrolysis process during the later stages of development. Cluster 7 (11 compounds) declined rapidly at stage 1–3, then stabilized, including mouthfeel-modulating 4-aminobutyric acid (GABA) [35]. These profiles inform targeted functional ingredient production from cap tissue.

In the stipe, 63 differential amino acids and derivatives were grouped into six clusters (Figure 3b) under the same criteria. Cluster 1 (Phe-Asp, Arg-Val, SAH) rose to a stage 3–5 peak, then declined; SAH exhibited tissue specificity (earlier peak, lower stipe levels), reflecting divergent metabolic demands of stipe structural maturation and cap spore development. Cluster 3 (eight compounds) rose at stage 1–3, declined at stage 3–7, then spiked at stage 7; tyrosine was lower in the stipe (except stage 1), and umami peptides (Tyr-Gly, Asp-Val, Met-Asp) were present [36]. Cluster 4 (taste-active free amino acids: glutamate, alanine) declined overall with lower stipe levels, confirming the cap as the primary taste-active metabolite reservoir. Clusters 6–7 showed contrasting patterns: Cluster 6 peaked at stage 3, dropped at stage 5, then rebounded (pyroglutamic acid was higher in an early stipe, lower in a late stipe); Cluster 7 (two compounds) rose to a stage 5 peak, then declined, with saccharopine showing a unique cross-tissue profile.

#### 3.3.2. Nucleosides, Nucleotides, and Analogs

Edible mushrooms are rich in nucleic acids that undergo enzymatic degradation to generate umami-enhancing nucleotides, which are key to their palatability and exert synergistic effects with free amino acids to further boost mushroom taste [7]. Figure 3a shows 19 cap-derived differential nucleosides, nucleotides and analogs clustered into seven groups, with stage-specific expression patterns linked to energy metabolism and oxidative stress regulation. Cluster 1 (uridine 5′-diphosphate, 5′-UDP) decreased at stages 1–3, then increased, indicating stage-specific roles in energy metabolism and nucleic acid biosynthesis during key developmental transitions. Cluster 2 (six DAMs) declined stepwise; nicotinamide adenine dinucleotide phosphate (NADP) mediates energy status and reactive oxygen species (ROS) scavenging, while flavin mononucleotide (FMN) and flavin adenine dinucleotide (FAD) act as core redox cofactors [37,38]. Cluster 3 (reduced nicotinamide adenine dinucleotide, NADH; inosine; adenosine) peaked at stage 3, reflecting elevated metabolic activity for critical physiological energy demands [39,40]. Clusters 4–5 (six DAMs) peaked at stage 5, including umami-contributing uridine 5′-monophosphate (5′-UMP) [12]. Cluster 6 (nicotinamide riboside, NR; UDP-xylose) was stable at stages 1–5, then upregulated, with NR (a NAD precursor) regulating taste-active compound biosynthesis and UDP-xylose modulating cell wall texture, implying a late developmental metabolic shift [41,42]. Cluster 7 (guanosine diphosphate fucose, GDP-fucose) declined sharply at stages 1–3 then stabilized; the dynamics of GDP-fucose may reflect altered cell wall integrity or host–pathogen interactions [43].

Figure 3b identifies 20 stipe-derived differential nucleosides, nucleotides and analogs in six clusters, with clear tissue-specific abundance and expression differences vs. the cap, revealing distinct stipe energy allocation and metabolic regulation. Cluster 1 (two DAMs) increased to a stage 3–5 plateau, then declined; FAD levels were consistently lower in the stipe, indicating tissue-specific energy demands and higher cap redox activity. Cluster 3 (inosine) peaked at stage 3, decreased, then rose sharply at stage 7 (stipe > cap at stage 7, comparable at stages 1–5). Cluster 4 (three DAMs) declined stepwise; adenosine was higher in the stipe only at stage 1, reflecting reduced mature stipe nucleotide synthesis vs. sustained cap activity. Cluster 5 (NADP) declined steeply at stages 1–3, then gradually, with consistent stipe under-abundance (peak at stage 1 in both tissues) indicating early redox activity and cap-prioritized NADP-dependent processes. Cluster 6 (2′-Deoxyguanosine 5′-monophosphate, dGMP) rose at stages 1–3, dropped at stages 3–5, then rebounded, regulating nucleic acid metabolism and oxidative stress [44]. Cluster 7 (12 DAMs) peaked at stage 5; 5′-UDP was consistently higher in the stipe (except stage 1) for structural polysaccharide synthesis, and UDP-xylose was stipe-dominant at stages 3–5 (for elongation) and cap-dominant at stages 1/7 (for reproductive tissue development).

#### 3.3.3. Organic Acids and Derivatives

Organic acids are key to food taste, nutrition retention and shelf-life extension, act as essential energy sources for mushrooms, and interact with basic taste attributes as critical taste-active and aroma precursor compounds that shape food sensory quality [45]. Figure 3a shows 24 cap-derived differential organic acids and derivatives clustered into seven groups, with stage-specific expression reflecting metabolic and taste regulatory dynamics. Cluster 1 (jasmonic acid, homocitric acid, 2-methylcitrate) decreased at stages 1–3, then increased: jasmonic acid modulates disease resistance and taste, and homocitric acid (TCA intermediate) links energy metabolism to umami and tartness [46,47]. Cluster 2 (four DAMs) declined stepwise, including sourness-contributing lactate, taste balance-regulating 3-hydroxypropionic acid, and mevalonate pathway/heme synthesis intermediates (mevalonic acid 5-pyrophosphate/coproporphyrin III) associated with taste development and oxidative stress [48,49,50,51]. Cluster 3 (uric acid) peaked at stage 3. Clusters 4–5 (three DAMs total) peaked at stage 5, featuring lipid oxidation-inhibiting medium-chain hydroxy acids that serve as flavor intermediates [52]. Cluster 6 (α-ketoglutaric acid, α-ketoisovaleric acid) was stable at stages 1–5, then upregulated: the former (TCA intermediate) modulates umami as a glutamate precursor; the latter influences bitterness, with their late surge supporting reproductive maturation and flavor biosynthesis post early biomass accumulation [53,54]. Cluster 7 (11 DAMs) declined sharply at stages 1–3, then stabilized; citric acid enhances flavor complexity and pyruvate acts as a pungency precursor, with the trend reflecting early growth energy diversion and maturation-phase metabolic homeostasis [55,56].

Figure 3b identified 16 stipe-derived differential organic acids and derivatives in three clusters, with tissue-specific abundance patterns revealing divergent metabolic and functional priorities vs. the cap. Cluster 2 (six DAMs) declined to stage 3, rose to stage 5, then slightly decreased: fumaric/maleic acid were consistently lower in the stipe (cap prioritizes sourness for spore dispersal, stipe for structural stability), and α-ketoglutaric acid (higher in stipe except stage 7, peak at stage 1) supports sustained stipe elongation energy demands. Cluster 5 (seven DAMs) declined sharply at stages 1–3, then gradually reduced; mevalonic acid 5-pyrophosphate was lower in the stipe, except at stage 7, indicating cap-dominated flavor/aroma development and late stipe surge for structural/defensive needs. Cluster 7 (three DAMs) rose to a stage 5 peak, then declined; 2-hydroxybutyric acid aligned with prior findings (lower stipe levels at stages 1–3, higher at 5–7), with early cap dominance linked to reproductive readiness and late stipe accumulation supporting structural reinforcement [11].

#### 3.3.4. Saccharides

Soluble sugars and polyols, key for food sweetness and bitter taste masking, have concentrations modulated by mushroom species and pre-/post-harvest processing [12]. In the cap, 17 differential saccharides formed six clusters (Figure 3a): D-sedoheptulose and 6-phosphogluconic acid (Cluster 1) decreased at stages 1–3, then increased, while nine glycolysis-related phosphorylated sugar DAMs (fructose/mannose/glucose/trehalose derivatives) in Cluster 7 dropped sharply at stages 1–3, then stabilized, supporting fungal growth and sporulation via ATP production [57].

In the stipe, 16 differential saccharides clustered into six groups (Figure 3b). Seven Cluster 1 DAMs rose to a plateau at stages 3–5, then declined. Phosphorylated sugars (fructose/mannose/glucose derivatives) were more abundant in the stipe at stages 3–7 (cap-dominant only at stage 1), consistent with the stipe’s higher fiber and carbohydrate content, reflecting their dual roles as energy substrates and cell wall synthesis precursors [58]. Cluster 5 saccharides declined steeply, then gradually; α, α’-trehalose 6-phosphate showed similar dynamics in both tissues with lower stipe levels. Cluster 7 (gluconic acid, D-sedoheptulose, perseitol) peaked at stage 5, then declined: gluconic acid was abundant in both tissues (stipe > cap at stage 5); D-sedoheptulose was stipe-dominant across all stages; perseitol was stipe-exclusive. The stage 5 peak may indicate a metabolic shift for fruiting body maturation/stress preparation, with spatial distribution revealing specialized functions: the stipe stores D-sedoheptulose and perseitol, while the cap reserves gluconic acid for immediate energy demands.

These stage-specific metabolic changes directly imply predictable differences in consumer sensory experience. At stage 3 (Closed cup), the highest levels of umami-active glutamate and S-adenosylmethionine (a key precursor of sulfur-containing volatiles) should produce strong umami taste and intense characteristic mushroom aroma, matching the preferred fresh flavor of commercial mushrooms. Previous studies have confirmed that major aroma volatiles, including dimethyl disulfide (DMDS) and dimethyl trisulfide (DMTS), are most abundant at similar closed-cap stages in edible mushroom, supporting the link between our non-volatile data and real flavor perception [16,59]. At stage 5 (Cup 2), reduced umami compounds and increased levels of bitter amino acids (e.g., tyrosine) and astringent peptides likely result in lower umami intensity, noticeable bitterness, and a less delicate mouthfeel. These metabolic-sensory correlations provide a practical basis for recommending stage 3 as the optimal harvest time for high-flavor *A. bisporus.* To intuitively visualize the tissue-specific accumulation differences in all identified non-volatile taste-related metabolites between cap and stipe at stage 3 (the optimal harvest stage proposed in this study), Supplementary Figure S8 was constructed with Z-score normalized heatmaps covering four core taste metabolite categories (Figure S8a–d). Consistent with the above statistical analysis, most key umami substances, sulfur-containing aroma precursors and antioxidant metabolites (including glutamate, ergothioneine, S-adenosylmethionine, inosine and citric acid) exhibited obviously higher accumulation levels in C3 cap tissue. By contrast, phosphorylated hexose saccharides displayed predominant enrichment in S3 stipe, which was consistent with the tissue-specific carbohydrate allocation for stipe structural development. The visual evidence from Figure S8 further supports our inference that stage 3 (closed cup) mushroom caps contain the richest pool of taste-determining non-volatile metabolites.

Moreover, numerous endogenous bioactive substances present in *A. bisporus* exerted synergistic interactions to modulate its umami intensity and holistic taste profile. Amino acids, nucleosides, nucleotide, organic acids, and a range of other metabolites collectively contributed to the formation of umami sensation while harmonizing the overall taste composition. Consistent with this taste-regulating mechanism, a recent study further verified that fruit and vegetable juices differing in acidity and inherent flavor traits can differentially reshape the taste properties of algal materials via modulating their intrinsic umami attributes [60]. In addition, different cooking methods altered mushroom aroma via Maillard reaction, lipid oxidation and Strecker degradation. Baking generated the greatest number of flavor volatiles. 1-octen-3-one was the primary aroma compound with an ultra-high odor activity value (OAV). Heat produced various aldehydes, benzyl alcohol, sulfur volatiles and 2-methylpyrazine, while C8 alcohols were thermally unstable. SAM acted as a key precursor for sulfur aroma synthesis during cooking [61]. Collectively, these results highlighted that developmental stage and tissue localization regulate saccharide, amino acid, nucleoside, nucleotide and organic acid metabolism, with implications for optimizing mushroom cultivation and the targeted extraction of taste-modifying and bioactive compounds.

### 3.4. KEGG Enrichment Analysis of DAMs in Cap and Stipe

**Figure 4:**
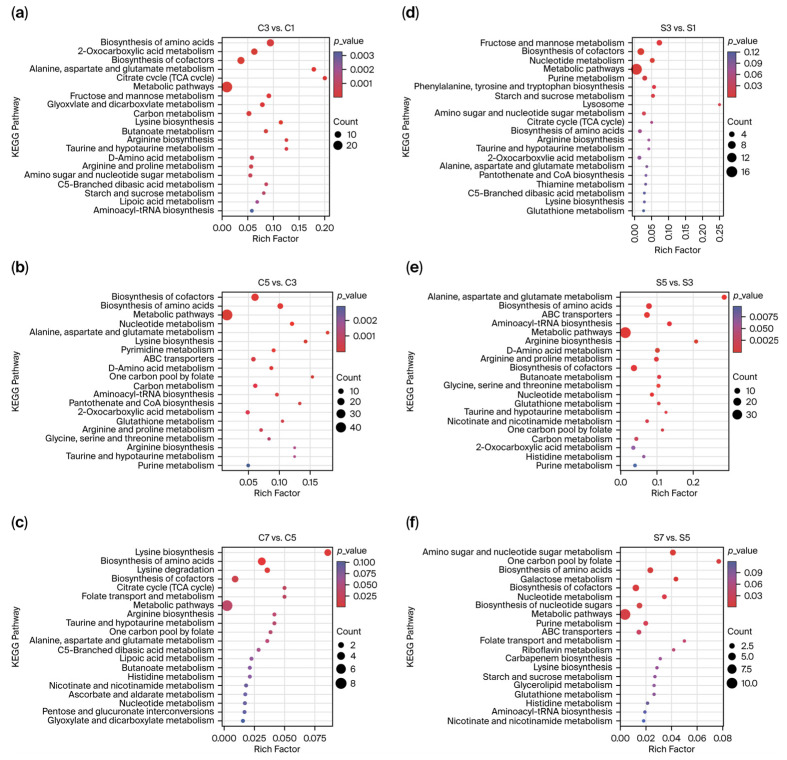
Enrichment analysis of KEGG metabolic pathway regulating non-volatile taste-related DAMs during fruiting body development: (**a**) C3 vs. C1; (**b**) C5 vs. C3; (**c**) C7 vs. C5; (**d**) S3 vs. S1; (**e**) S5 vs. S3; (**f**) S7 vs. S5.

KEGG enrichment analysis identified core pathways of taste-related metabolites across *A. bisporus* fruiting body development (Figure 4). In cap tissue, C3 vs. C1 DAMs enriched in amino acid/cofactor biosynthesis, 2-oxocarboxylic acid/alanine–aspartate–glutamate metabolism, TCA cycle and metabolic pathways; C5 vs. C3 in cofactor/amino acid biosynthesis, nucleotide/alanine–aspartate–glutamate metabolism, lysine biosynthesis and metabolic pathways; and C7 vs. C5 in lysine biosynthesis/degradation, amino acid/cofactor biosynthesis. Amino acid biosynthesis (abv01230), cofactor biosynthesis (abv01240) and lysine biosynthesis (abv00300) were persistently enriched in cap development. In stipe tissue, S3 vs. S1 DAMs linked to fructose–mannose metabolism, cofactor/nucleotide/purine metabolism and metabolic pathways; S5 vs. S3 in alanine–aspartate–glutamate metabolism, amino acid/cofactor biosynthesis, ABC transporters, aminoacyl-tRNA biosynthesis and metabolic pathways; S7 vs. S5 in amino-nucleotide sugar metabolism, one carbon pool by folate, amino acid/cofactor biosynthesis, and galactose/nucleotide metabolism. Amino acid biosynthesis (abv01230), cofactor biosynthesis (abv01240) and nucleotide metabolism (abv01232) were continuously regulated in stipes, underpinning their key roles in flavor formation during ontogeny.

**Figure 5:**
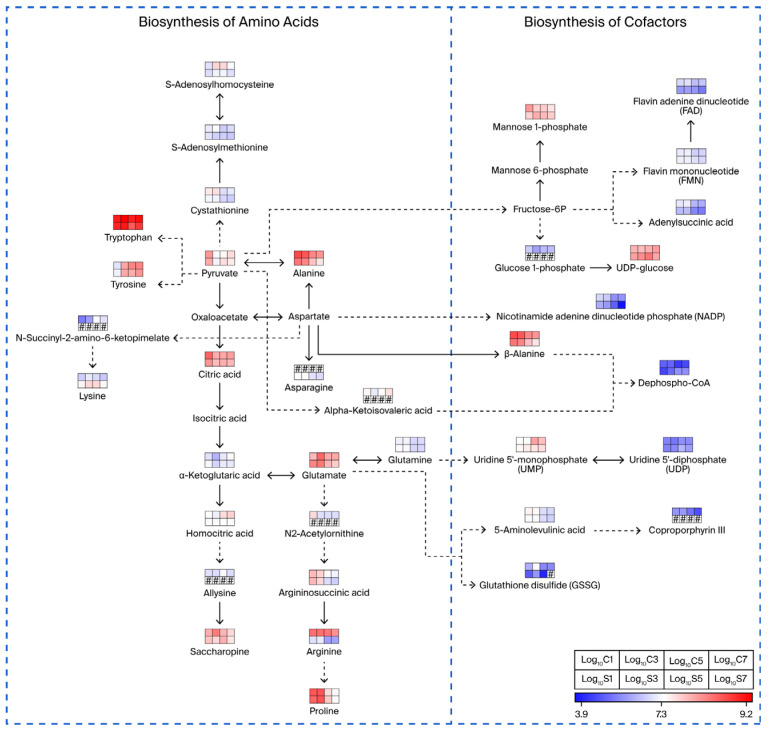
Changes in taste-related DAMs mapped to metabolic pathways during the fruiting body development of *A. bisporus*. This map was developed based on the KEGG pathway and relevant studies. The colored boxes in front of each metabolite represent log_10_C1, log_10_C3, log_10_C5, log_10_C7, log_10_S1, log_10_S3, log_10_S5, and log_10_S7 values according to the color scale. Solid arrows indicate direct generation and dashed arrows indicate indirect generation. The symbol # indicates that the metabolite was undetected.

Figure 5 delineates these pathways, highlighting dominant amino acid and cofactor biosynthesis; pathway-enriched metabolites and their stage-specific peaks are marked with colored boxes. Pyruvate, a central metabolic precursor, branches into multiple synthetic pathways, with levels declining after stage 1 in both tissues [62]. Pyruvate transamination generates alanine, which remained high at stages 1/3, slightly decreased at stages 5/7, and was more abundant in caps [63]. For aspartate family amino acids, pyruvate converts to TCA intermediate oxaloacetate (precursor of aspartate, glutamate, lysine) [64]; glutamate and lysine peaked at stage 3 in both tissues, with lysine higher in stipes. Tryptophan (aromatic amino acid) is synthesized from pyruvate via a branched pathway (tryptophan synthase), remaining abundant across all stages [64,65]. Tyrosine is derived from pyruvate via the phenylalanine pathway, with levels rising rapidly from stage 1 and stabilizing after stage 3 [64]. Beyond protein synthesis, these amino acids mediate energy metabolism (alanine gluconeogenesis) and metabolic regulation (glutamate–glutamine nitrogen balance) [62,66].

Cofactors act as enzyme activation switches and energy carriers: FMN/FAD (core for redox reactions and energy metabolism) and NADP (involved in fatty acid synthesis and pentose phosphate pathway) showed gradual declines across all stages in both tissues [67,68]; UDP (energy currency for glycogen/nucleotide metabolism) peaked at stage 5 [69]. Consumed cofactors are regenerated via metabolic cycles (e.g., NADP redox cycle) to sustain metabolism [70]. Glycolysis links to glycogen metabolism (glucose 1-phosphate → UDP-glucose → glycogen) and TCA cycle (pyruvate → oxaloacetate → TCA). Pyruvate is the central hub connecting glycolysis and the TCA cycle, with its metabolic fate (oxidative catabolism/anabolism) dynamically regulated by cellular energy status (ATP/NAD levels) [71]. This network integrates amino acid/cofactor biosynthesis with glycolysis/TCA and regulatory signals, forming a closed loop for cellular homeostasis and environmental adaptation. Dynamic changes in these key metabolites during fruiting body development are critical for the unique taste of *A. bisporus*.

### 3.5. Differential Metabolomic Profiles Between Cap and Stipe Tissues

**Figure 6:**
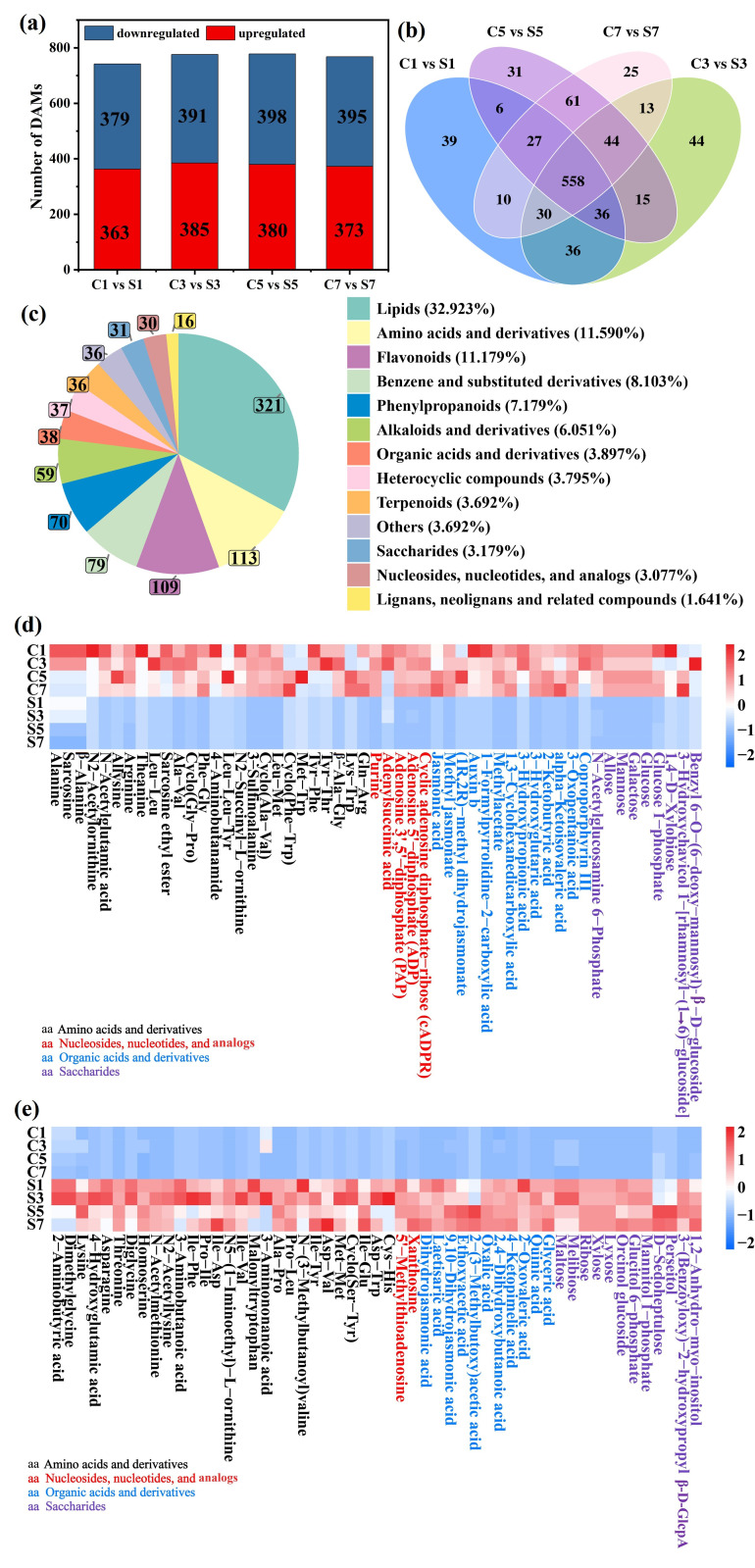
Metabolite profiles between the cap and stipe during the fruiting body development. (**a**) The numbers of up- and downregulated DAMs in four comparison groups (C1 vs. S1, C3 vs. S3, C5 vs. S5, and C7 vs. S7). (**b**) A Venn diagram depicting the number of DAMs in the four comparison groups. (**c**) A pie chart presenting the categories and corresponding ratios of the 975 DAMs. A heatmap showing that 53 (**d**) and 53 (**e**) taste-related DAMs were consistently higher in the cap and stipe during all growth stages.

Tissue type drove profound metabolic differentiation in *A. bisporus* beyond developmental stage effects (Figure 1c). DAM screening between cap and stipe at the same stage identified 975 total DAMs across four comparisons (C1/S1, C3/S3, C5/S5, C7/S7; Tables S8–S11), indicating tissue-specific factors exert a stronger metabolic influence than developmental progression alone. Cap and stipe metabolomic profiles differed consistently at the same stage (Figure 6a), with disparity rising slightly at stage 3 and stabilizing thereafter: 742 (363/379 up/down), 776 (385/391), 778 (380/398) and 768 (373/395) DAMs were detected in the four comparisons, respectively. A core set of 558 DAMs was shared across all groups, with 39, 44, 31 and 25 unique DAMs in C1/S1, C3/S3, C5/S5 and C7/S7 (Figure 6b). Of all DAMs, 11.59% (113) were amino acids/derivatives, 3.90% (38) organic acids/derivatives, 3.18% (31) saccharides, and 3.08% (30) nucleosides/nucleotides/analogs (Figure 6c). Notably, 53 taste-related DAMs were consistently more abundant in the cap (26 amino acids/derivatives, 5 nucleosides/nucleotides/analogs, 13 organic acids/derivatives, 9 saccharides; Figure 6d), and another 53 in the stipe (28 amino acids/derivatives, 2 nucleosides/nucleotides/analogs, 11 organic acids/derivatives, 12 saccharides; Figure 6e). This distinct tissue-specific metabolic divergence validates these compounds as potential biomarkers for tissue-specific selection in mushroom-based food development.

Combining all the above findings, this study provides practical guidance for the industrial cultivation, post-harvest processing and product development of *A. bisporus*. Harvesting mushrooms at the closed cup stage (stage 3) is highly recommended to acquire products with optimal taste and antioxidant benefits. The dynamic patterns of taste-related metabolites can be used as biochemical indicators to regulate temperature, humidity and carbon dioxide levels in mushroom houses during commercial factory cultivation. Furthermore, the obvious metabolic divergence between caps and stipes supports their targeted utilization: mushroom caps, which are rich in umami compounds and antioxidants, are well suited for fresh consumption and natural flavor production, whereas stipes with high carbohydrate content can be processed into deep-processed food, fermentation substrates and animal feed. From an economic perspective for commercial cultivation, the above practical applications bring substantial economic gains to growers and mushroom enterprises. Harvesting at the optimal stage effectively reduces defective products caused by poor flavor and inferior sensory quality, and elevates commodity grades and market prices. Precise environmental regulation based on metabolite characteristics stabilizes yield and quality, and cuts unnecessary energy consumption in factory mushroom houses, thus lowering production costs. The classified utilization of caps and stipes greatly improves the comprehensive utilization rate of raw materials, minimizes production waste and maximizes the overall economic profit of the whole cultivation and processing chain. In summary, this study provides a solid metabolic basis for the sustainable development and economic efficiency improvement of the *A. bisporus* cultivation industry.

## 4. Conclusions

Non-targeted metabolomics characterized four developmental stages of *A. bisporus*, identifying 1358 differential metabolites (lipids, amino acids/derivatives, organic acids/derivatives, nucleosides/nucleotides/analogs, saccharides, flavonoids, etc.) in cap and stipe. By VIP > 1, *p* < 0.01 and FC ≥ 2/≤ 0.5, 492 and 446 differential metabolites were detected in cap and stipe, respectively; taste-related metabolites in both tissues were clustered into seven groups via K-means. Comparative analysis revealed significant tissue-specific metabolic divergence: cap tissue had glutamate (core umami) and S-adenosylmethionine (aroma precursor) peaking at stage 3, suggesting a preliminary optimal harvest window (to be validated by sensory evaluation and EUC calculation), with antioxidants (glutathione disulfide, ergothioneine) showing parallel trends and forming the metabolic basis for this stage. Organic acids (citric acid, pyruvate) and nucleotides (NADP, FMN, FAD) accumulated more in immature tissues and declined with maturation. Stipe maintained high phosphorylated six-carbon sugars at stages 3–7 for structural growth, while cap only had transient dominance of these sugars at stage 1.

Pathway analysis showed taste-related metabolites regulate *A. bisporus* taste formation mainly via amino acid/cofactor/lysine biosynthesis and nucleotide metabolism. Moreover, 975 DAMs were identified between cap and stipe across stages; 53 taste-related compounds were consistently more abundant in the cap, with another 53 showing opposite tissue accumulation patterns. This study clarified dynamic metabolite and taste quality changes during *A. bisporus* fruiting body development, advanced understanding of its taste variation, and provided a scientific foundation for improving edible mushroom taste quality and strain breeding innovation. In practical industrial application, this study also offered actionable references for standardized harvesting, precise environmental control in factory cultivation, and classified deep processing of caps and stipes, which can effectively promote the economic benefits and comprehensive utilization rate of the *A. bisporus* industry. A key limitation of this study is that volatile compounds, which strongly determine mushroom flavor and aroma, were not analyzed. Future work combining non-volatile metabolomics, volatile profiling, and sensory testing will enable a more complete assessment of *A. bisporus* flavor quality.

## Data Availability

The original contributions presented in this study are included in the article/Supplementary Material. Further inquiries can be directed to the corresponding authors.

## Supplementary Materials

The following supporting information can be downloaded at: https://www.mdpi.com/article/10.3390/foods15132375/s1, Figure S1: Venn diagram showing the number of metabolites identified from caps and stipes; Figure S2: Pie plot showing the numbers and classification of the identified metabolites in cap and stipe tissues, respectively; Figure S3: The principal component analysis score plot of QC samples with first principal component; Figure S4: OPLS-DA score plot of (a) C3 vs. C1; (b) C5 vs. C3; (c) C7 vs. C5; (d) S3 vs. S1; (e) S5 vs. S3; (f) S7 vs. S5; Figure S5: The permutation analysis of the OPLS-DA model: (a) C3 vs. C1; (b) C5 vs. C3; (c) C7 vs. C5; (d) S3 vs. S1; (e) S5 vs. S3; (f) S7 vs. S5; Figure S6: Heat map of the top 20 differential accumulated metabolites that are significantly upregulated and downregulated for pairwise comparisons: (a) C3 vs. C1; (b) C5 vs. C3; (c) C7 vs. C5; (d) S3 vs. S1; (e) S5 vs. S3; (f) S7 vs. S5; Figure S7: The heatmap of shared differentially accumulated metabolites (DAMs) in cap (a) and stipe (b) during fruiting body development. Color from blue (low) to red (high) indicates the level of each metabolite; Figure S8: Heatmaps of Z-score normalized relative abundance of four categories of non-volatile taste-related metabolites in cap (C3) and stipe (S3) at stage 3. (a) Amino acids and derivatives; (b) Nucleosides, nucleotides, and analogues; (c) Organic acids and derivatives; (d) Saccharides. Each row represents one annotated taste-related metabolite, and each column corresponds to C3 and S3 samples. Color gradient from green to red indicates low to high standardized metabolite abundance based on Z-score; Table S1: The non-normalized peak areas data of each identified metabolite; Table S2: List of DAMs between C3 and C1; Table S3: List of DAMs between C5 and C3; Table S4: List of DAMs between C7 and C5; Table S5: List of DAMs between S3 and S1; Table S6: List of DAMs between S5 and S3; Table S7: List of DAMs between S7 and S5; Table S8: List of DAMs between C1 and S1; Table S9: List of DAMs between C3 and S3; Table S10: List of DAMs between C5 and S5; Table S11: List of DAMs between C7 and S7.
