# Supplementary material for: Non-Volatile Taste Profile Dynamics Across Developmental Stages of Agaricus bisporus Fruiting Bodies
Source: Foods. 2026 Jul 3;15(13):2375. doi: 10.3390/foods15132375 (PMC13360935; doi:10.3390/foods15132375)
Supplement: Supplementary file 1 [file foods-15-02375-s001.zip › Figure S8 (a).pdf]

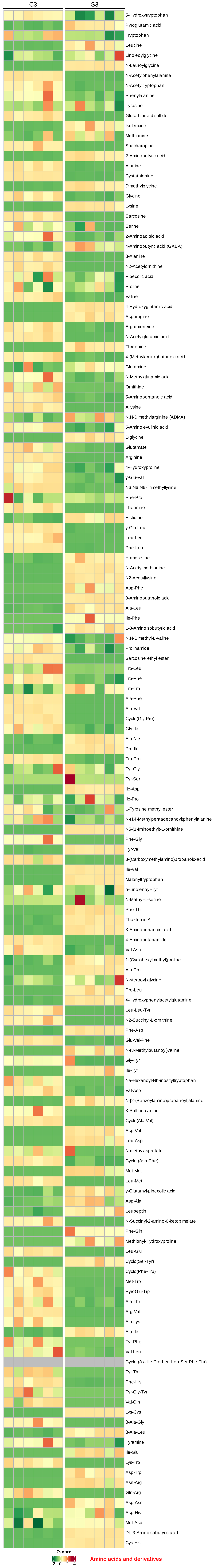
C3
S3
5-Hydroxytryptophan
Pyroglutamic acid
Tryptophan
Leucine
Linoleoylglycine
N-Lauroylglycine
N-Acetylphenylalanine
N-Acetyltryptophan
Phenylalanine
Tyrosine
Glutathione disulfide
Isoleucine
Methionine
Saccharopine
2-Aminobutyric acid
Alanine
Cystathionine
Dimethylglycine
Glycine
Lysine
Sarcosine
Serine
2-Aminoadipic acid
4-Aminobutyric acid (GABA)
β-Alanine
N2-Acetylornithine
Pipecolic acid
Proline
Valine
4-Hydroxyglutamic acid
Asparagine
Ergothioneine
N-Acetylglutamic acid
Threonine
4-(Methylamino)butanoic acid
Glutamine
N-Methylglutamic acid
Ornithine
5-Aminopentanoic acid
Allysine
N,N-Dimethylarginine (ADMA)
5-Aminolevulinic acid
Diglycine
Glutamate
Arginine
4-Hydroxyproline
γ-Glu-Val
N6,N6,N6-Trimethyllysine
Phe-Pro
Theanine
Histidine
γ-Glu-Leu
Leu-Leu
Phe-Leu
Homoserine
N-Acetylmethionine
N2-Acetyllysine
Asp-Phe
3-Aminobutanoic acid
Ala-Leu
Ile-Phe
L-3-Aminoisobutyric acid
N,N-Dimethyl-L-valine
Prolinamide
Sarcosine ethyl ester
Trp-Leu
Trp-Phe
Trp-Trp
Ala-Phe
Ala-Val
Cyclo(Gly-Pro)
Gly-Ile
Ala-Nle
Pro-Ile
Trp-Pro
Tyr-Gly
Tyr-Ser
Ile-Asp
Ile-Pro
L-Tyrosine methyl ester
N-(14-Methylpentadecanoyl)phenylalanine
N5-(1-Iminoethyl)-L-ornithine
Phe-Gly
Tyr-Val
3-(Carboxymethylamino)propanoic-acid
Ile-Val
Malonyltryptophan
α-Linolenoyl-Tyr
N-Methyl-L-serine
Phe-Thr
Thaxtomin A
3-Aminononanoic acid
4-Aminobutanamide
Val-Asn
1-(Cyclohexylmethyl)proline
Ala-Pro
N-stearoyl glycine
Pro-Leu
4-Hydroxyphenylacetylglutamine
Leu-Leu-Tyr
N2-Succinyl-L-ornithine
Phe-Asp
Glu-Val-Phe
N-(3-Methylbutanoyl)valine
Gly-Tyr
Ile-Tyr
Na-Hexanoyl-Nb-inosityltryptophan
Val-Asp
N-[2-(Benzoylamino)propanoyl]alanine
3-Sulfinoalanine
Cyclo(Ala-Val)
Asp-Val
Leu-Asp
N-methylaspartate
Cyclo (Asp-Phe)
Met-Met
Leu-Met
γ-Glutamyl-pipecolic acid
Asp-Ala
Leupeptin
N-Succinyl-2-amino-6-ketopimelate
Phe-Gln
Methionyl-Hydroxyproline
Leu-Glu
Cyclo(Ser-Tyr)
Cyclo(Phe-Trp)
Met-Trp
PyroGlu-Trp
Ala-Thr
Arg-Val
Ala-Lys
Ala-Ile
Tyr-Phe
Val-Leu
Cyclo (Ala-Ile-Pro-Leu-Leu-Ser-Phe-Thr)
Tyr-Thr
Phe-His
Tyr-Gly-Tyr
Val-Gln
Lys-Cys
β-Ala-Gly
β-Ala-Leu
Tyramine
Ile-Glu
Lys-Trp
Asp-Trp
Asn-Arg
Gln-Arg
Asp-Asn
Asp-His
Met-Asp
DL-3-Aminoisobutyric acid
Cys-His
Zscore
-2 0 2 4
Amino acids and derivatives
