# Supplementary material for: Non-Volatile Taste Profile Dynamics Across Developmental Stages of Agaricus bisporus Fruiting Bodies
Source: Foods. 2026 Jul 3;15(13):2375. doi: 10.3390/foods15132375 (PMC13360935; doi:10.3390/foods15132375)
Supplement: Supplementary file 1 [file foods-15-02375-s001.zip › Figure S8 (c).pdf]

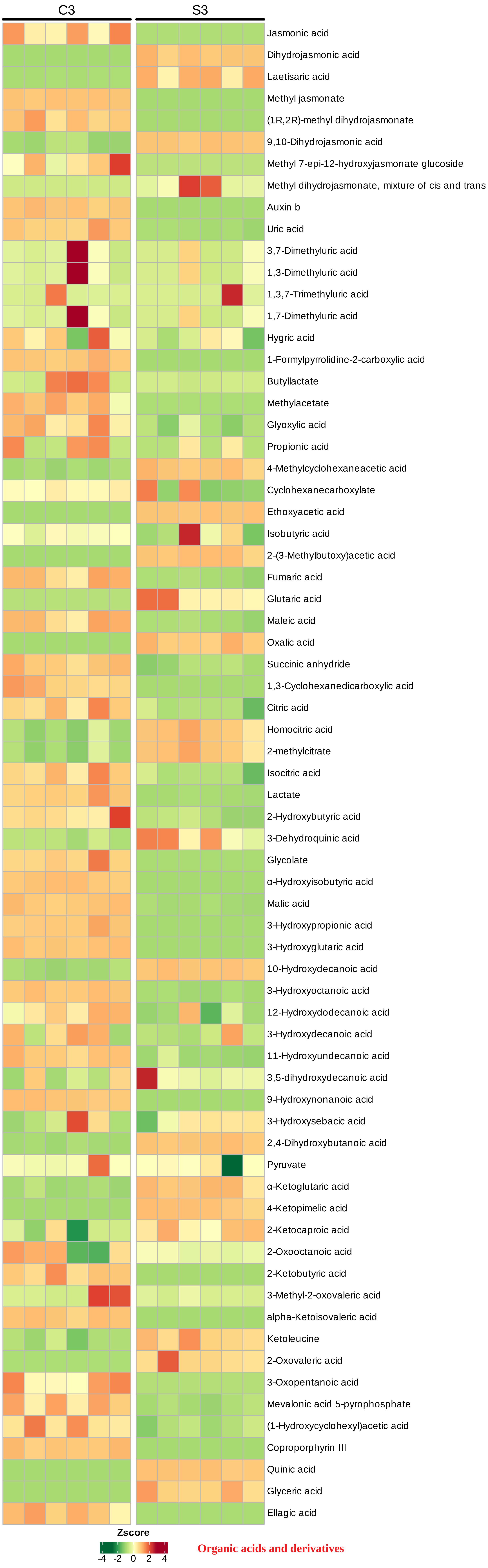
C3
S3
Jasmonic acid
Dihydrojasmonic acid
Laetisaric acid
Methyl jasmonate
(1R,2R)-methyl dihydrojasmonate
9,10-Dihydrojasmonic acid
Methyl 7-epi-12-hydroxyjasmonate glucoside
Methyl dihydrojasmonate, mixture of cis and trans
Auxin b
Uric acid
3,7-Dimethyluric acid
1,3-Dimethyluric acid
1,3,7-Trimethyluric acid
1,7-Dimethyluric acid
Hygric acid
1-Formylpyrrolidine-2-carboxylic acid
Butyllactate
Methylacetate
Glyoxylic acid
Propionic acid
4-Methylcyclohexaneacetic acid
Cyclohexanecarboxylate
Ethoxyacetic acid
Isobutyric acid
2-(3-Methylbutoxy)acetic acid
Fumaric acid
Glutaric acid
Maleic acid
Oxalic acid
Succinic anhydride
1,3-Cyclohexanedicarboxylic acid
Citric acid
Homocitric acid
2-methylcitrate
Isocitric acid
Lactate
2-Hydroxybutyric acid
3-Dehydroquinic acid
Glycolate
α-Hydroxyisobutyric acid
Malic acid
3-Hydroxypropionic acid
3-Hydroxyglutaric acid
10-Hydroxydecanoic acid
3-Hydroxyoctanoic acid
12-Hydroxydodecanoic acid
3-Hydroxydecanoic acid
11-Hydroxyundecanoic acid
3,5-dihydroxydecanoic acid
9-Hydroxynonanoic acid
3-Hydroxysebacic acid
2,4-Dihydroxybutanoic acid
Pyruvate
α-Ketoglutaric acid
4-Ketopimelic acid
2-Ketocaproic acid
2-Oxooctanoic acid
2-Ketobutyric acid
3-Methyl-2-oxovaleric acid
alpha-Ketoisovaleric acid
Ketoleucine
2-Oxovaleric acid
3-Oxopentanoic acid
Mevalonic acid 5-pyrophosphate
(1-Hydroxycyclohexyl)acetic acid
Coproporphyrin III
Quinic acid
Glyceric acid
Ellagic acid
Zscore
-4 -2 0 2 4
Organic acids and derivatives
