# Supplementary material for: Non-Volatile Taste Profile Dynamics Across Developmental Stages of Agaricus bisporus Fruiting Bodies
Source: Foods. 2026 Jul 3;15(13):2375. doi: 10.3390/foods15132375 (PMC13360935; doi:10.3390/foods15132375)
Supplement: Supplementary file 1 [file foods-15-02375-s001.zip › Supplementary Information.pdf]

Electronic Supplementary Information

Non-volatile taste profile dynamics across developmental stages of *Agaricus bisporus* fruiting bodies

Lingzhong Wan 1,†, Hongjuan Wang 1,2,†, Sheng Liu 3, Ying Ni 1, Xiaonan Deng 1, Xiaoming Yan 1, Changjiu Tian 1,2,

Qianwen Li 4,* and Jiabao Zhu 1,*

1 Institute of Industrial Crops, Anhui Academy of Agricultural Sciences, Hefei 230001, China;

wan520lz@mail.ustc.edu.cn (L.W.); whj14725@163.com (H.W.); ny18895329564@163.com (Y.N.);

xn_deng@foxmail.com (X.D.); 19909699660@163.com (X.Y.); changjiutian@163.com (C.T.)

2 Edible and Medicinal Mushroom Innovation Centre, Anhui Academy of Agricultural Sciences,

Hefei 230001, China

3 College of Food Science and Engineering, Shandong Agriculture and Engineering University,

Jinan 250100, China; z2023066@sdaeu.edu.cn

4 School of Materials and Chemistry, Anhui Agricultural University, Hefei 230036, China

* Correspondence: liqianwen@ahau.edu.cn (Q.L.); wlz_3513@foxmail.com (J.Z.); Tel./Fax: +86-551-62825125 (J.Z.)

† These authors contributed equally to this work.

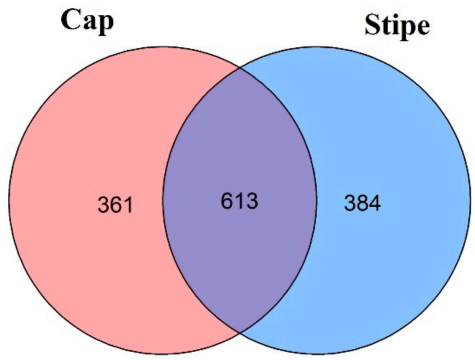

Figure S1. Venn diagram showing the number of metabolites identified from caps and stipes.

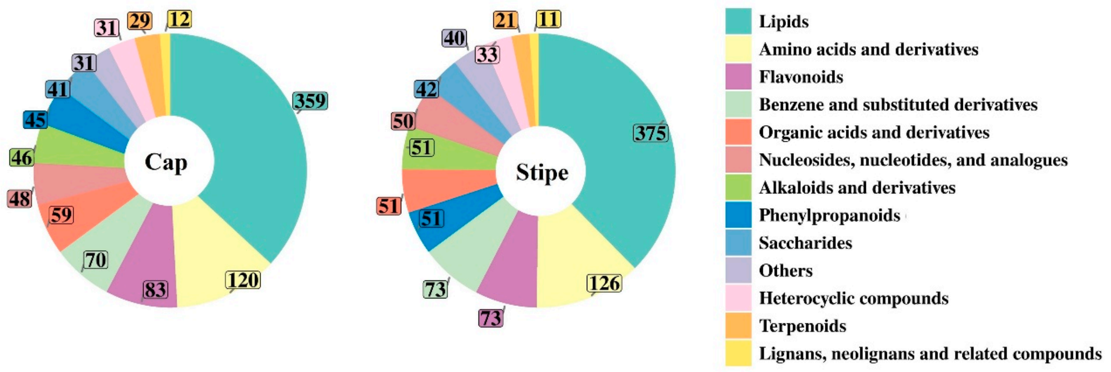

Figure S2. Pie plot showing the numbers and classification of the identified metabolites in cap and stipe tissues, respectively.

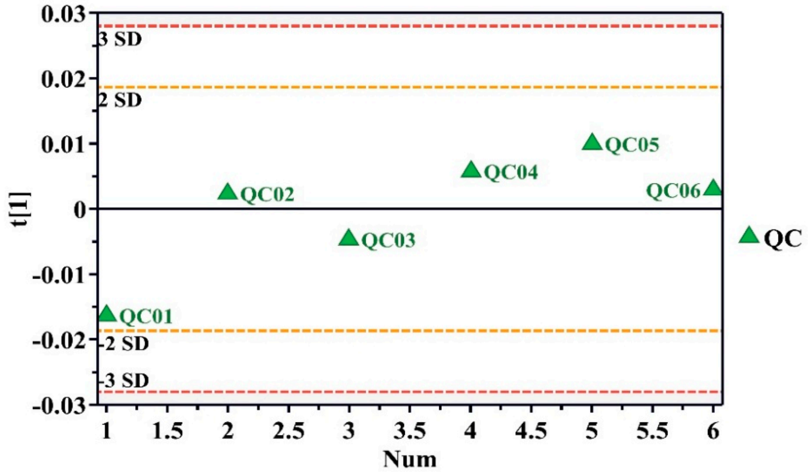

Figure S3. The principal component analysis score plot of QC samples with first principal component.

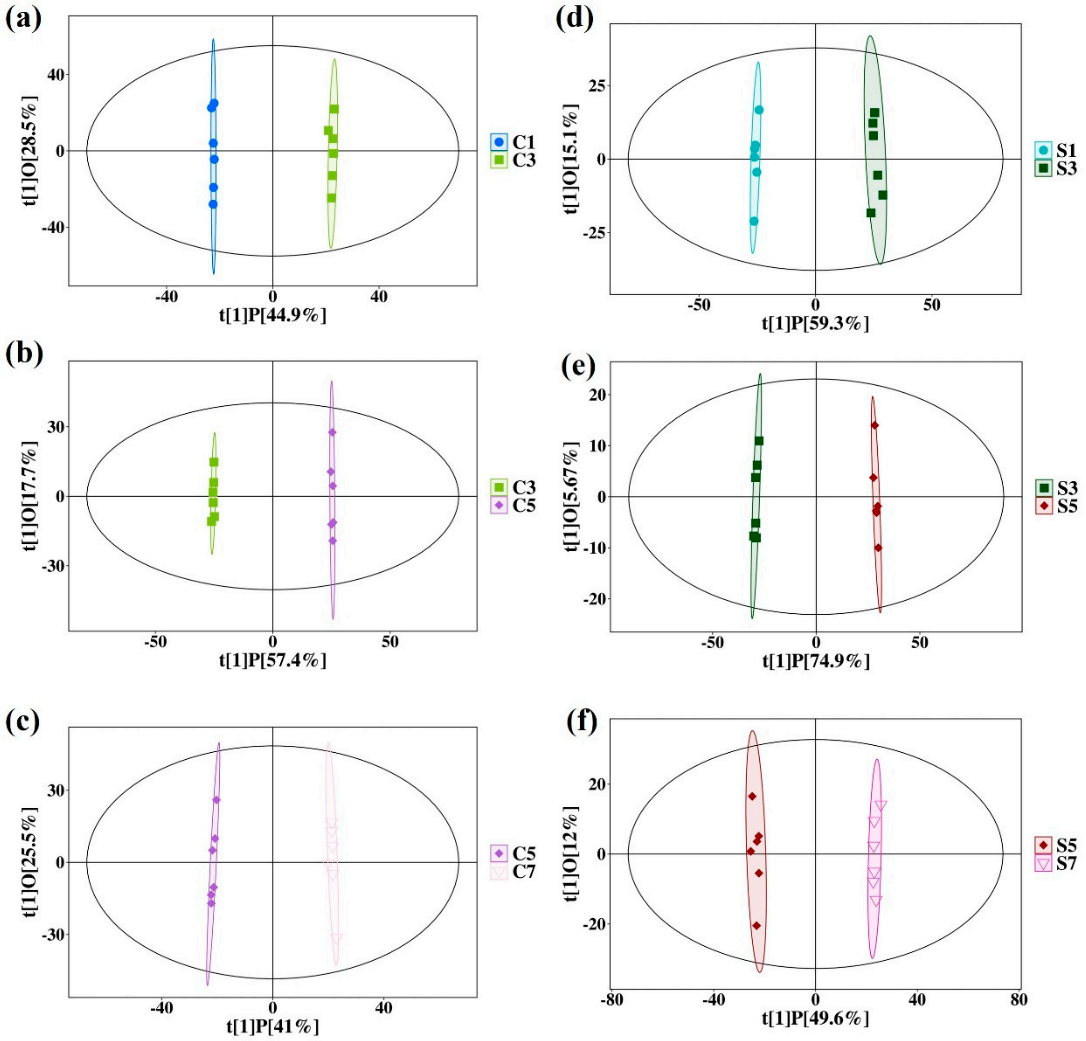

Figure S4. OPLS-DA score plot of (a) C3 vs C1; (b) C5 vs C3; (c) C7 vs C5; (d) S3 vs S1; (e) S5 vs S3; (f) S7 vs S5.

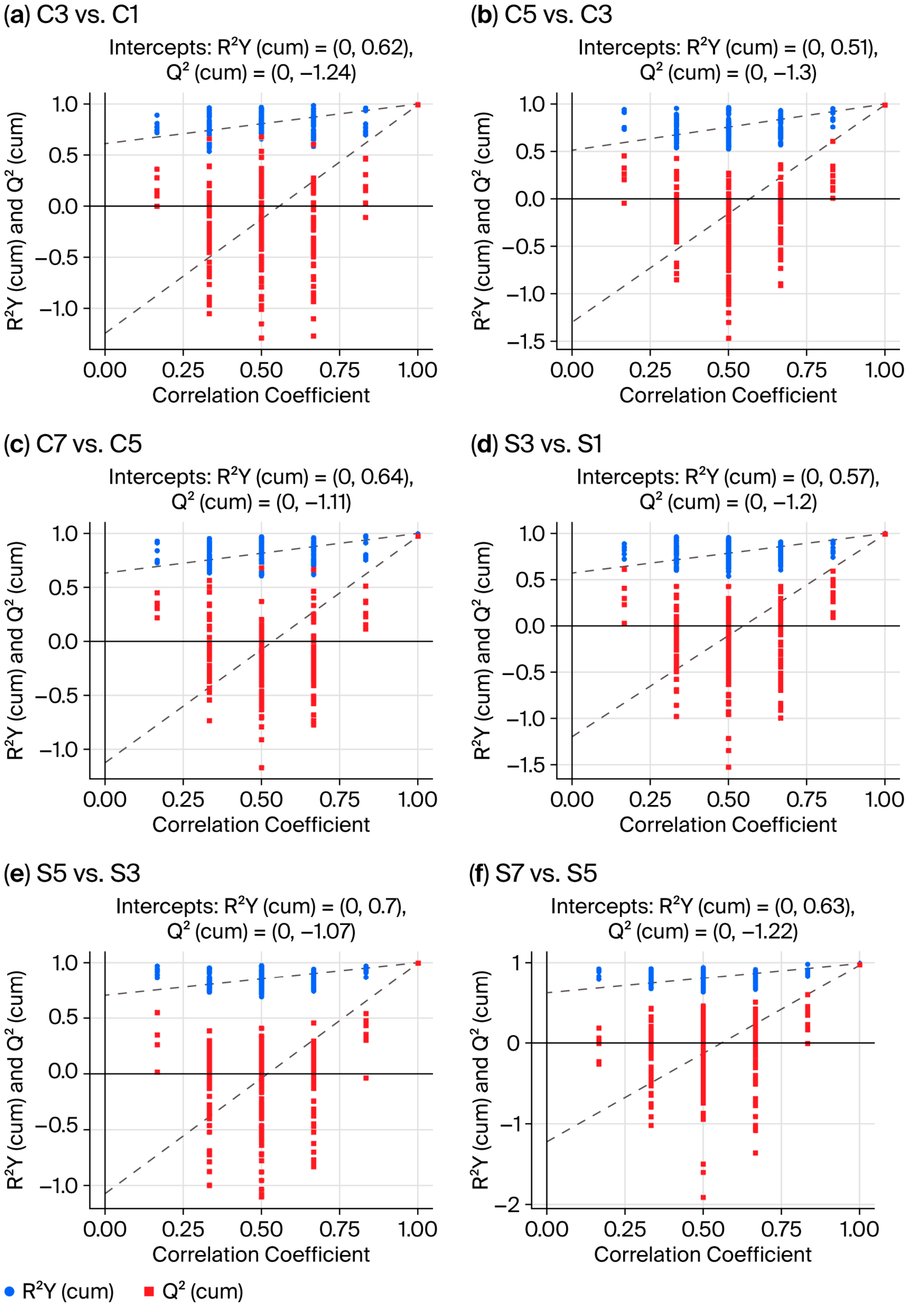

Figure S5. The permutation analysis of OPLS-DA model: (a) C3 vs C1; (b) C5 vs C3; (c) C7 vs C5; (d) S3 vs S1; (e) S5 vs S3; (f) S7 vs S5.

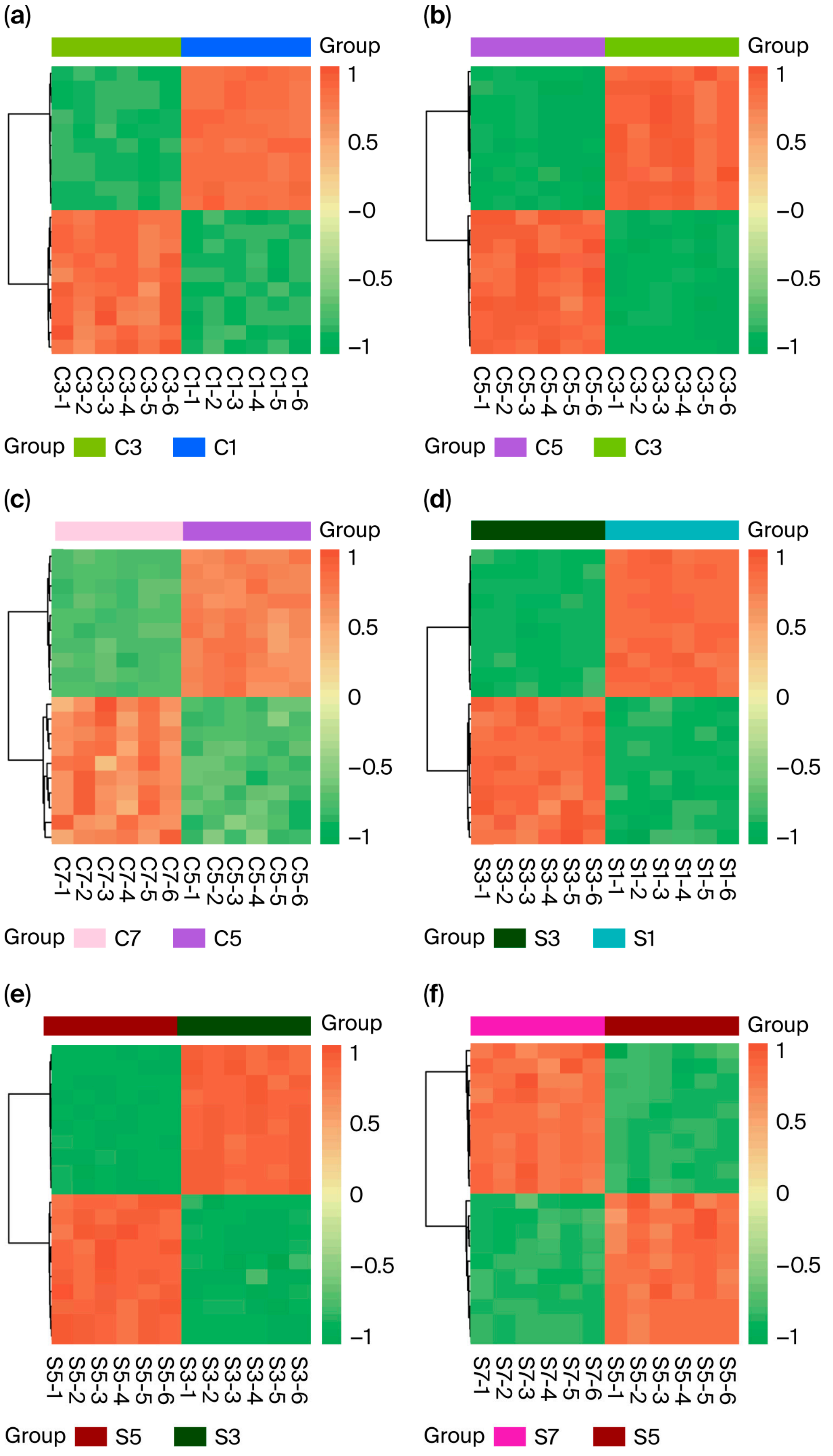

Figure S6. Heat map of the top 20 differential accumulated metabolites that are significantly

upregulated and downregulated for pairwise comparisons：(a) C3 vs C1; (b) C5 vs C3; (c) C7 vs C5; (d) S3 vs S1; (e) S5 vs S3; (f) S7 vs S5.

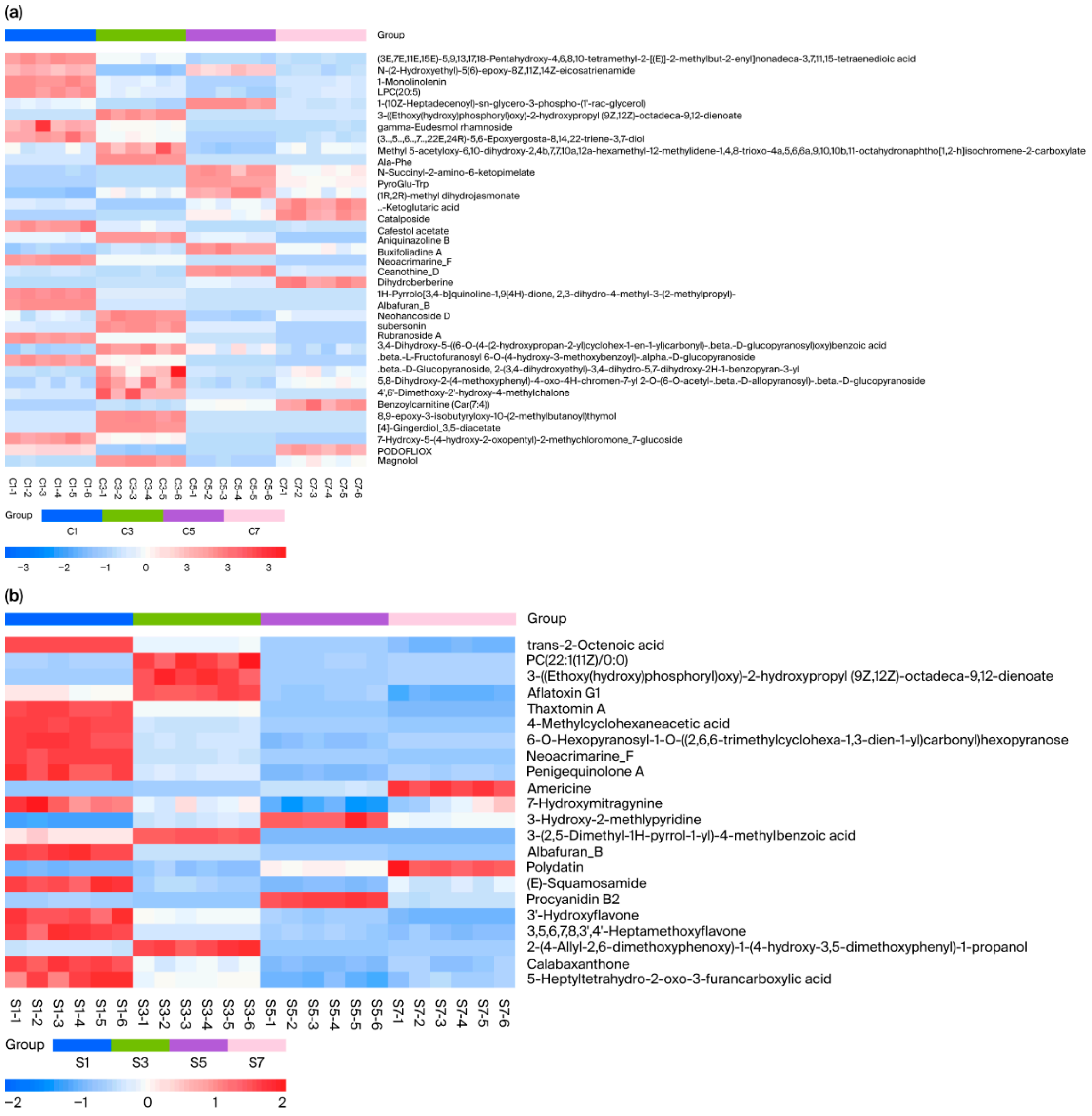

Figure S7. The heatmap of shared differentially accumulated metabolites (DAMs) in cap (a) and stipe (b) during fruiting body development. Color from blue (low) to red (high) indicates the level of each metabolite.

Figure S8. Heatmaps of Z-score normalized relative abundance of four categories of non-volatile taste-related metabolites in cap (C3) and stipe (S3) at stage 3. (a) Amino acids and derivatives; (b) nucleosides, nucleotides, and analogs; (c) organic acids and derivatives; (d) saccharides. Each row represents one annotated taste-related metabolite, and each column corresponds to C3 and S3 samples. Color gradient from green to red indicates low to high standardized metabolite abundance based on Z-score.

(The figures are provided as PDF files in the supplementary attachments due to their large size.)
