# Supplementary figures and images for: Non-Volatile Taste Profile Dynamics Across Developmental Stages of Agaricus bisporus Fruiting Bodies
Source: Foods. 2026 Jul 3;15(13):2375. doi: 10.3390/foods15132375 (PMC13360935; doi:10.3390/foods15132375)

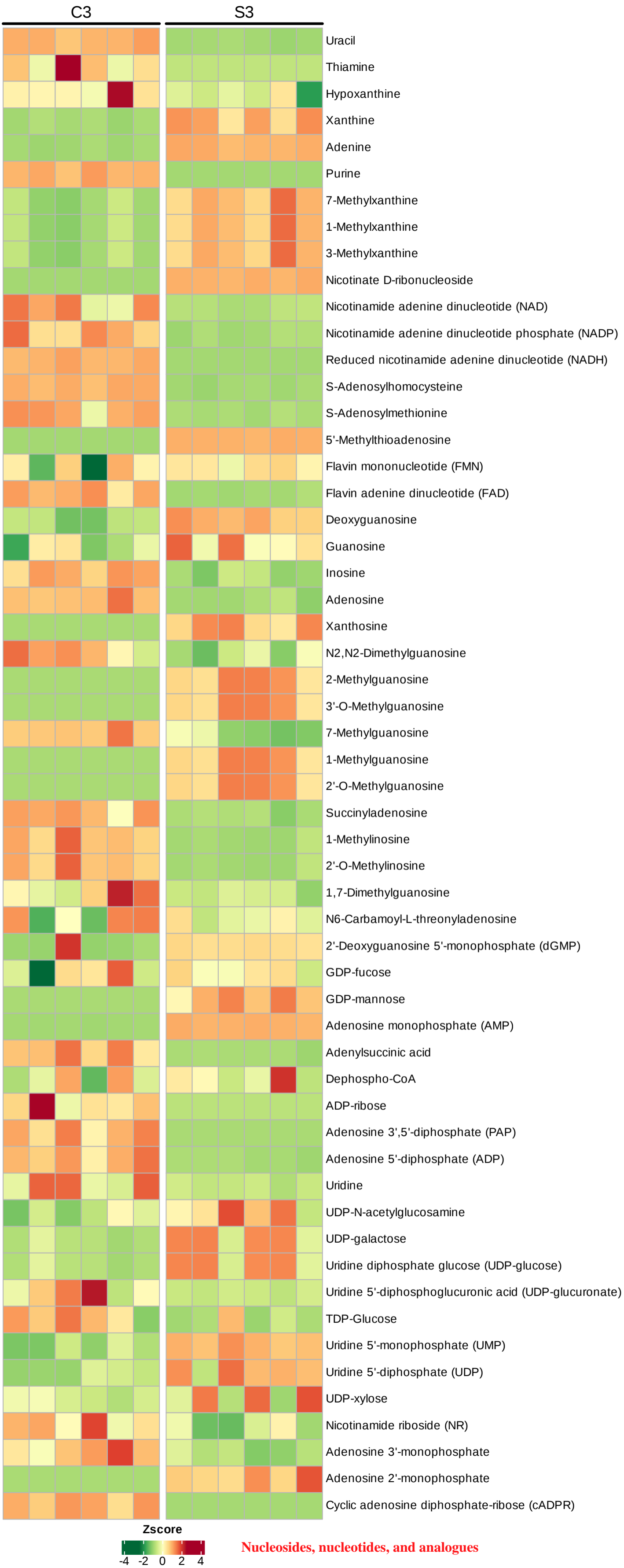

Supplement: Supplementary file 1 [file foods-15-02375-s001.zip › Figure S8 (b).pdf]

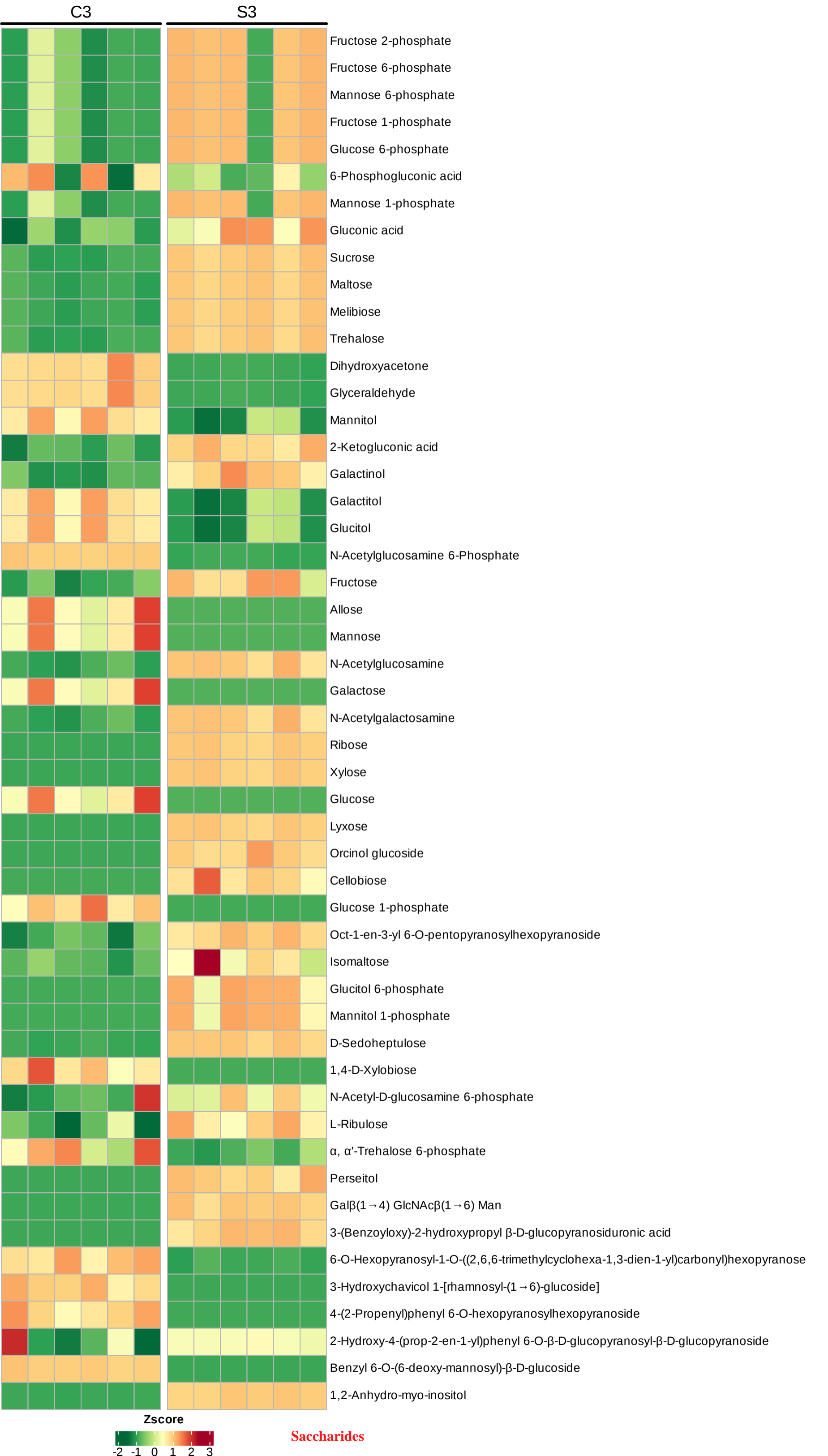

Supplement: Supplementary file 1 [file foods-15-02375-s001.zip › Figure S8 (d).pdf]
